# Food safety knowledge, attitudes and practices of institutional food-handlers in Ghana

**DOI:** 10.1186/s12889-016-3986-9

**Published:** 2017-01-06

**Authors:** Fortune Akabanda, Eli Hope Hlortsi, James Owusu-Kwarteng

**Affiliations:** Department of Applied Biology, Faculty of Applied Sciences, University for Development Studies, P. O. Box 24, Navrongo Campus, Ghana

**Keywords:** Food-handlers, Food safety, Ghana, Hygienic practices

## Abstract

**Background:**

In large scale cooking, food is handled by many individuals, thereby increasing the chances of food contamination due to improper handling. Deliberate or accidental contamination of food during large scale production might endanger the health of consumers, and have very expensive repercussions on a country. The purpose of this study was to evaluate the food safety knowledge, attitudes, and practices among institutional food- handlers in Ghana.

**Methods:**

The study was conducted using a descriptive, cross-sectional survey of 29 institutions by conducting face to face interview and administration of questionnaire to two hundred and thirty-five (235) institutional food-handlers. The questionnaire was peer-reviewed and pilot tested in three institutions in the Upper East Region of Ghana, before the final version was distributed to food-handlers. The questionnaire was structured into five distinctive parts to collect information on (i) demographic characteristics, (ii) employees’ work satisfaction, (iii) knowledge on food safety, (iv) attitudes towards food safety and (v) food hygiene practices.

**Results:**

Majority of the food-handlers were between 41–50 years (39.1%). Female respondents were (76.6%). In our study, the food-handlers were knowledgeable about hygienic practices, cleaning and sanitation procedures. Almost all of the food-handlers were aware of the critical role of general sanitary practices in the work place, such as hand washing (98.7% correct answers), using gloves (77.9%), proper cleaning of the instruments/utensils (86.4%) and detergent use (72.8%). On disease transmission, the results indicates that 76.2% of the food- handlers did not know that *Salmonella* is a food borne pathogens and 70.6% did not know that hepatitis A is a food borne pathogen. However, 81.7% handlers agreed that typhoid fever is transmitted by food and 87.7% agreed that bloody diarrhea is transmitted by food. Logistic regression analysis testing four models showed statistically significant differences (*p* < 0.05), for models in which the explanatory variable was the level of education.

**Conclusions:**

In generally, the institutional food-handlers have satisfactory knowledge in food safety but this does not translate into strict hygienic practices during processing and handling food products.

## Background

When food is cooked on a large scale, it may be handled by many individuals and thus increasing the chances of contamination of the final food. Unintended contamination of food during large scale cooking, leading to food-borne disease outbreaks can pose danger to the health of consumers and economic consequence for nations [1–3].

Food-borne related illnesses have increased over the years, and negatively affected the health and economic well-being of many developing nations [4]. The World Health Organization (WHO) states that about 1.8 million persons died from diarrheal diseases in 2005, mainly due to the ingestion of contaminated food and drinking water. Food poisoning occurs as a result of consuming food contaminated with microorganisms or their toxins, the contamination arising from inadequate preservation methods, unhygienic handling practices, cross-contamination from food contact surfaces, or from persons harboring the microorganisms in their nares and on the skin [5, 6]. Unhygienic practices during food preparation, handling and storage creates the conditions that allows the proliferation and transmission of disease causing organisms such as bacteria, viruses and other food-borne pathogens [7, 8]. Additionally, many reported cases of food-borne viral diseases have been attributed to infected food-handlers involved in catering services [9].

In Ghana, both public and private institutions often have food service or catering units where meals are served to both staff and clients. Such institutions may include schools, research institutes, hospitals and prisons. To prevent outbreak of food-borne diseases in these institutions, high standards of hygienic and safety practices by food-handlers are essential parts of an overall food safety program implemented by these institutions. Although institutional food-handlers may possess the required knowledge and skills needed in food safety practice, errors due to human handling are often cited in several food-borne disease outbreaks [10–12]. As Greig et al. [11] reports, about 97% of reported food poisoning cases are due to the improper handling of foods by persons involved in catering services.

The knowledge, attitudes and practices of food-handlers have been reported in studies from different countries around world [13–18]. This is because a combination the three factors: knowledge, attitude and practice of food-handlers, play dominant role in food safety with regards to food service industry [19]. In Ghana, previous studies have evaluated the knowledge, attitudes and practices of food-handlers in selected hotels in Accra [2], and food hygiene practices by street food vendors [20]. Recently, food safety knowledge, attitudes and self-reported practices of food handlers in institutional foodservice in Accra-Ghana has also been reported [21]. All these studies were however limiting in scope as they were restricted to only Accra, the capital city. At the moment, there is no published report on the knowledge, attitude and practice of food-handlers in institution selected from different geographical regions of Ghana. Such studies are however, important as they provide a nation-wide assessment of training needs, attitudinal changes and effectiveness of training and education to provide continuous consumer assurance of the safety of food. Such investigations will also provide better understanding of the interactions of prevailing food safety knowledge, attitudes and practices of food-handlers throughout the country, Ghana.

This study therefore sought to assess the knowledge, attitudes, and practices of institutional food-handlers in Ghana, with regard to food hygienic practices and over-all safety.

## Methods

### Study population

**Fig. 1:**
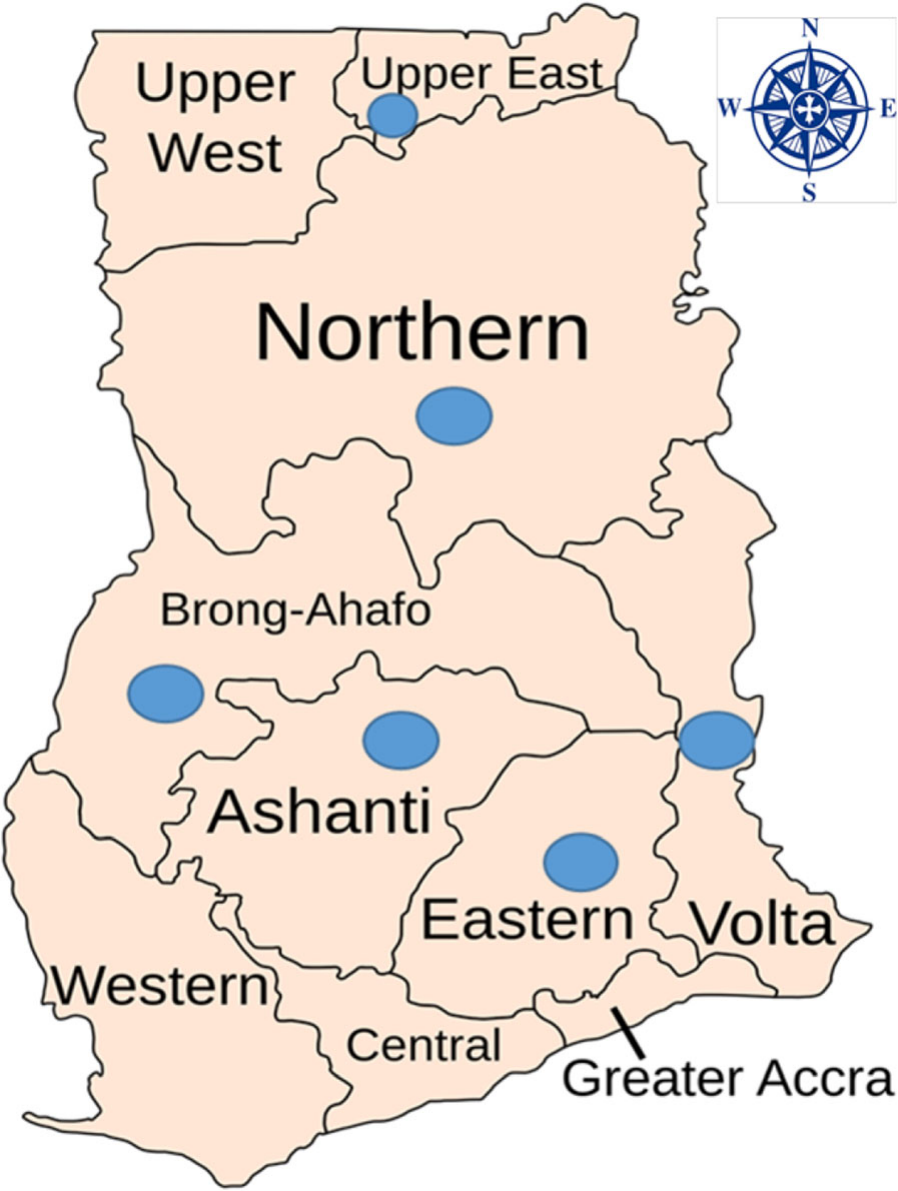
Administrative regions of Ghana. Blue dot-circles indicate the regions from which institutional food handlers were sampled for the studies

A total of 235 institutional food-handlers participated in the study. A descriptive, cross-sectional survey of 29 institutions was employed in this study. The institutions included ten (10) Senior High Schools, nine (9) District Hospitals, two (2) Prison Services, six (6) Universities/Polytechnics and one (1) Health Research Center located in 5 administrative regions including Upper East, Northern, Ashanti, Volta, Brong-Ahafo and Eastern regions of Ghana (Fig 1).

### Interviews

Face-to-face interviews were conducted using structured questionnaire to collect information on the knowledge, attitudes and practices of the food-handlers on food safety. The questionnaire was peer-reviewed and pilot tested in three institutions in the Upper East Region of Ghana, before a final version was administered to food-handlers. Participants were interviewed by the researchers and trained research assistants using the structured questionnaire. Data was collected between December 2014 and June 2015.

The questionnaire was structured into five distinctive sections. Section one was to collect information on respondents’ demographic characteristics such as gender, age, level of education and length of employment in the food service business. Section two was concerned with information on the employees’ work satisfaction while sections 3, 4 and 5 were concerned with information on employees’ knowledge of food safety, attitude towards food safety and food hygiene practices respectively.

Questions pertaining to demographic information of respondents and employees’ work satisfaction were adapted from Soares et al. [22] and Jevšnik et al. [16]. Answers were graded on a scale of five, with 1 indicating “strongly disagree” and five indicating “strongly agree”. Questions on food safety knowledge, attitudes and practices were adapted from previously published works [13, 22–24].

The section of questionnaire dealing with food safety knowledge comprised 25 close-ended questions with three possible answers; “true”, “false”, and “do not know”. These questions specifically dealt with respondents’ knowledge of personal hygiene, cross contamination, food-borne diseases, microorganisms, temperature control and hygienic practices. A scale ranging between 0 and 24 (representing the total number of questions on food safety knowledge) was used to evaluate the overall knowledge of respondents. Food-handlers that obtained total score ≤16 points were considered to have “insufficient” knowledge and those that had scores ≥17 points (≥68% accuracy) were considered to have “good” knowledge of food safety.

Questions pertaining to attitudes (section 4) were aimed at determining the understanding of food-handlers about food safety. Here, attitudes was used to mean “a complex mental state involving beliefs, feelings, values and dispositions to act in certain ways” [19]. This section had 20 statements/questions that required three possible responses: “agree”, “disagree”, and “don’t know or remember”. For evaluation, food-handlers that answered to 13 or less statements/questions correctly were measured to have “insufficient or poor” understanding, whereas those that responded to 14 or more statements/questions correctly were measured to have “good” understanding.

In section five, which dealt with food hygiene practices, the good hygienic practices of respondents (institutional food-handlers) were assessed and evaluated based on self-reporting of personal hygiene and other safe food handling practices. The section had 11 statements/questions with two possible responses: “yes”, and “no”. Each correct practice reported scored one (1) point. For evaluation, a score ≥ 70% by an individual respondent was considered as having “good” food hygienic practice.

### Statistical analyses

The statistical package for social science (SPSS) for Windows (version 11.0, 2001, Chicago, IL) was used for analyses of data. Difference in means were considered statistically significant at *p* < 0.05. Mean scores of responses were converted into percentages for purposes of easy interpretation of results. One way analysis of variance (ANOVA) was used to compare the mean scores of knowledge, attitude, and practice among the different educational levels of respondents. The mean score of knowledge, attitude, and practice were compared by *t*-test. The responses were also reclassified into two categorical responses ‘yes’ and ‘no’ for correct and incorrect responses respectively.

In order to identify the variables that impacted on the observed results, four logistic regression analysis models were developed. The four models included knowledge of foodborne diseases, knowledge and attitudes regarding personal hygiene, knowledge and attitudes regarding temperature control, and knowledge and attitudes towards food hygienic practices. Predictive variables including age, length of employment and education level were incorporated into all models with general knowledge on food safety as the dependent variable.

## Results and discussion

### Demographic characteristics

Table 1 presents the summarized demographic profile of respondents in this study. Out of the 235 food-handlers who participated in this study, 76.6% (*n* = 180) were female while 23.4% (*n* = 55) were males. Most studies have reported higher proportion of females [22, 25, 26] involvement in food handling. A greater number (39.1%) of the participants were in the age bracket of 41–50 years with average age of 41.5 ± 9.5 years. Similar studies showed that workers in older age brackets had better hygiene scores than their younger colleagues [27, 28]. Approximately 14.5% of the participants in this study did not have any formal education. However, a previous study demonstrated that irrespective of educational level, employees performance in food safety knowledge was not satisfactory and thus a cause for public concern [29]. A greater number of participants (76.2%) had >5 years of experience working in the foodservice industry with an average length of 11 ± 7.8 years. Eighty three percent were full time workers in the institutions whiles the rest were part time workers. Only a few (8.1%) of the respondents self-reported that they have ever received training in food safety. Although several studies have shown that training may contribute to upgrading the food safety knowledge of food-handlers, this does not always translate into a positive change in food handling behavior and attitudes [12, 30].

**Table 1:** Demographic characteristics of institutional food-handlers in Ghana (*n* = 235)

Characteristics	Number	Percent	Mean ± SD	Range
Gender				
Female	180	76.6		
Male	55	23.4	N/A	N/A
Age (years)				
21–30	18	7.7		
31–40	78	33.2		
41–50	92	39.1		
51–60	47	20.0	41.5 ± 9.5	22–60
Marital status				
Single	24	10.2		
Married	161	68.5		
Divorced	19	8.1		
Widow/widower	31	13.2		
Education				
No formal education	34	14.5		
Basic	91	38.7		
JHS/JSS	57	24.3		
SHS/SSS	13	5.5		
Post-secondary/Vocational	22	9.4		
Tertiary	18	7.6		
Length of employment (years)				
<5	56	23.8		
5–10	61	25.9		
11–20	109	46.4		
21–30	9	38.3	11 ± 7.8	0.5–30
Employment status				
Full-time	197	83.8		
Part-time	38	16.2		
Food safety training course				
Yes	19	8.1		
No	216	91.9		

### Employees’ work satisfaction

Work satisfaction was surveyed to ascertain institutional food-handler’s satisfaction with the conditions, work load, work relations and other people reaction toward them (Table 2). In general, 24.7% of the institutional food-handlers affirmed that if they had to choose a profession again, they would choose the same profession, 48.5% said they would not choose the same profession, whiles 26.8% were indifferent about the issue of choosing a profession again.

**Table 2:** Employees’ work satisfaction

Statement/question	Response % (n)		
	Yes	No	Don’t know
If you could choose a profession, would you choose this same profession?	24.7 (58)	48.5 (114)	26.8 (63)
When you have personal trouble, do you share with your colleagues?	60.0 (141)	40.0 (94)	0 (0)
When you have personal trouble, did you share with your head of department?	64.3 (151)	35.7 (84)	0 (0)
Would you leave this work if you are offered something better at another place?	95.3 (224)	0.0 (0)	4.7 (11)
Is the work load adequate?	92.3 (217)	7.7 (18)	0 (0)
Is the kitchen staff respected by other workers of the institution?	14.9 (35)	85.1 (200)	0 (0)
Does the workplace provide all the necessary conditions to guaranteeing food safety?	62.1 (146)	37.9 (89)	0 (0)
Do the meals served present health risks to the people?	0 (0)	100.0 (235)	0 (0)

In answering the question; would you quit this work if you are offered something better at a different place, about 95.3% affirmed they will leave while 4.7% were not certain. About 62.1% said the workplace make available all the necessary conditions for guaranteeing food safety. On the issue of sharing problems, 64.3% agreed that they do share their personal problems with their head of departments.

### Food safety knowledge of food-handlers

In our study, the food-handlers were knowledgeable about hygiene practices, cleaning and sanitation procedures (Table 3). Majority of food-handlers in this study knew the importance of general sanitary practices such as regular hand washing at the work place (98.7% correct answers), wearing of gloves (77.9% correct answers), proper cleaning (86.4% correct answers) and detergent use (72.8%).

**Table 3:** Food safety knowledge of institutional food-handlers in Ghana

Statement	Response % (n)		
	Correct	Incorrect	Don’t know/remember
Washing hands before work reduces the risk of food contamination.	98.7 (232)	0.0 (0)	1.3 (3)
Using gloves while handling food reduces the risk of food contamination.	77.9 (183)	17.4 (41)	4.7 (11)
Proper cleaning and sanitization of utensils increase the risk of food contamination.	4.7 (11)	86.4 (203)	8.9 (21)
Eating and drinking at the work place increase the risk of food contamination.	25.1 (59)	60.9 (143)	14.0 (33)
Food prepared in advance reduces the risk of food contamination.	21.3 (50)	68.1 (160)	10.6 (25)
Reheating cooked foods can contribute to food contamination.	11.5 (27)	70.6 (166)	17.9 (42)
Washing utensils with detergent leaves them free of contamination.	72.8 (171)	26.8 (63)	0.4 (1)
Children, healthy adults, pregnant women and older individuals are at equal risk for food poisoning.	60.9 (143)	37.9 (89)	1.2 (3)
Typhoid fever can be transmitted by food.	81.7 (192)	13.6 (32)	4.7 (11)
AIDS can be transmitted by food.	8.5 (20)	86.8 (204)	4.7 (11)
Bloody diarrhea can be transmitted by food.	87.7 (206)	8.1 (19)	4.2 (10)
Salmonella is among the food-borne pathogens.	18.3 (43)	5.5 (13)	76.2 (179)
Hepatitis A virus is among the food-borne pathogens.	11.5 (27)	17.9 (42)	70.6 (166)
Swollen cans may contain the microorganism, *Clostridium botulinum*, which causes botulism.	13.6 (32)	10.2 (24)	76.2 (179)
Microbes are on the skin, in the nose and mouth of healthy food handlers.	71.5 (168)	25.1 (59)	3.4 (8)
Clean is the same as sanitized.	60.0 (141)	33.6 (79)	6.4 (15)
Cross contamination is when microorganisms from a contaminated food are transferred by the food handler’s hands or kitchen utensils to another food.	4.7 (11)	8.9 (21)	86.4 (203)
The correct temperature for storing perishable foods is 5 °C.	33.6 (79)	6.4 (15)	60.0 (141)
Hot, ready-to-eat food should be kept at a temperature of 65 °C.	60.0 (141)	6.4 (15)	33.6 (79)
Freezing kills all the bacteria that may cause food-borne illness.	59.1 (139)	39.2 (92)	1.7 (4)
Contaminated foods always have some change in color, odor or taste.	60.4 (142)	39.2 (92)	0.4 (1)
Raw vegetables are at higher risk of contamination than undercooked beef	40.9 (96)	28.9 (68)	30.2(71)
During infectious disease of the skin, it is necessary to take leave from work.	93.6 (220)	0.0 (0)	6.4 (15)
The health status of workers should be evaluated before employment.	99.6 (234)	0.4 (1)	0.0 (0)
The ideal place to store raw meat in the refrigerator is on the bottom shelf.	40.9 (96)	28.9 (68)	30.2(71)

The awareness of such important hygienic procedures by majority of the institutional food-handlers in this study is very appropriate. This is because the hands of food-handlers can serve as vectors in the spread of foodborne diseases due to poor personal hygiene or cross-contamination [14, 31]. Proper hand washing by food-handlers has been reported to significantly decrease the threat of diarrheal disease in child care facilities [32] and can therefore be encouraged as it could similarly help minimize the risk of diarrhea and other foodborne diseases in similar institutions. Despite a self-reported hand washing practiced by food-handlers, many employees’ in a study by Stepanović et al. [33] had coagulase-positive staphylococci isolated from their hands, and this could be a source of food contamination. Therefore, it is prudent to combine proper hand washing with the wearing of gloves and other hygienic practices in order to minimize the risk of contamination during food handling [34].

Regarding foodborne disease transmission, 76.2% of the food-handlers (respondents) did not know or remember that *Salmonella* is a food-borne pathogens and 70.6% did not know/remember that hepatitis A is a foodborne pathogen. On the other, 81.7 and 87.7% of respondents agreed that typhoid fever and bloody diarrhea respectively can be transmitted by food. The majority (86.8%) of respondents disagreed that HIV/AIDS is transmitted by food, which is an indication that public education on HIV/AIDS by the Ghana AIDS commission could be yielding results. These results support recently published work where majority of the respondents did not know if *Salmonella*, hepatitis A and B viruses, and *Staphylococcus* caused foodborne diseases [13, 22]. Over ninety percent (90%) of respondents agreed that taking leave from work in periods of infectious skin disease was necessary (Table 3). Additionally, 71.5% knew that microorganisms can be found on the skin and in the mouth and nose of healthy looking individuals. They also recognize that the health status of food-handlers should be assessed prior to employment.

On the other hand, food-handlers were less familiar with time and temperature abuse and its effect on food safety (Table 3). Anon [35] reported that improper handling of food, including the abuse of time-temperature, account for most food-borne disease outbreak. In this study, respondents had insufficient knowledge on time-temperature controls. This result is supported by others [14, 29] whose report show that knowledge of critical temperatures were insufficient amongst food-handlers. Similar findings on the lack of adequate knowledge on temperature controls by food-handlers have also been reported from different countries [36–38].

### Food safety attitudes of food-handlers

A reduction in the incidence of food-borne illnesses is strongly influenced by the attitudes of food-handlers towards the implementation of food safety plans. Thus, there is a strong linkage between positive behavior, attitudes and education of food-handlers in maintaining safe food handling practices [12].

Table 4 shows the attitudes of the food-handlers toward the prevention and control of food-borne diseases. About 60% of respondents indicated that using caps, masks, protective gloves and proper clothing can minimize the risk of food contamination, which is a positive attitude reported by majority of the respondents. Similarly, majority of respondents (93.6%) agreed that knives and cutting boards should be properly sanitized to prevent cross contamination of foods. Respondents also agreed that individuals with abrasions or cuts on their fingers or hands should not touch unwrapped foods (87.2%). The majority (88.1%) of food-handlers were aware that food should not be handled with long and painted fingernails. They were also mindful of the fact that dish towels could cross-contaminate foods (71.5%) and that well-cooked foods are free of contamination (86.4%). Thus, the general attitudes of the food-handlers toward food safety was satisfactory, except on issues relating to refrozen of defrosted food. About 81.7% of food-handlers had unsatisfactory attitude towards defrosted and refrozen foods. Refreezing a completely thawed food can present a serious health risk, as this process leads faster growth of contaminating bacteria. Freezing food only slows bacterial growth and does not necessarily kill the pathogens [39]. Respondents (86.4%) did not find it necessary to check the temperatures of refrigerators and freezers periodically (86.4%).

**Table 4:** Food safety attitudes of institutional food-handlers

Statement	Response % (n)		
	Agree	Disagree	Don’t know/remember
Well-cooked foods are free of contamination	86.4 (203)	8.9 (21)	4.7 (11)
Proper hand hygiene can prevent food-borne diseases.	93.6 (220)	0.0 (0)	6.4 (15)
When cleaning products are closed, they can be stored with cans and jars of food that are also closed.	28.9 (68)	30.2(71)	40.9 (96)
Raw and cooked foods should be stored separately to reduce the risk of food contamination.	40.9 (96)	28.9 (68)	30.2(71)
It is necessary to check the temperature of refrigerators/freezers periodically to reduce the risk of food contamination.	33.6 (79)	8.9 (21)	86.4 (203)
Defrosted foods should not be refrozen.	13.6 (32)	81.7 (192)	4.7 (11)
The health status of workers should be evaluated before employment.	60.0 (141)	6.4 (15)	33.6 (79)
The best way to thaw a chicken is in a bowl of cold water.	87.7 (206)	8.1 (19)	4.2 (10)
Wearing masks is an important practice to reduce the risk of food contamination.	60.0 (141)	6.4 (15)	33.6 (79)
Wearing gloves is an important practice to reduce the risk of food contamination.	60.0 (141)	6.4 (15)	33.6 (79)
Wearing caps and adequate clothing is an important practice to reduce the risk of food contamination.	60.0 (141)	6.4 (15)	33.6 (79)
Safe food handling is an important part of my job responsibilities	40.9 (96)	28.9 (68)	30.2 (71)
Learning more about food safety through training courses is important to me	30.2 (71)	28.9 (68)	40.9 (96)
Beards could contaminate food with foodborne pathogens	13.6 (32)	10.2 (24)	76.2(179)
Long and painted fingernails could contaminate food with foodborne pathogens.	88.1 (207)	11.9 (28)	0 (0)
Food handlers can be a source of foodborne outbreaks	64.3 (151)	35.7 (84)	0 (0)
Eggs must be washed immediately after delivery.	24.7 (58)	48.5 (114)	26.8 (63)
Dish towels can be a source of food contamination.	71.5 (168)	25.1 (59)	3.4 (8)
Knives and cutting boards should be properly sanitized to prevent cross contamination.	93.6 (220)	0 (0)	6.4 (15)
Food handlers who have abrasions or cuts on their hands should not touch foods without gloves.	87.2 (207)	0 (0)	12.8 (30)

### Food safety practices by food-handlers

Table 5 shows the food safety practices by institutional food-handlers. In assessing the food safety practices of the institutional food-handlers, 88.1% reported that they do not use gloves during the distribution of unpackaged foods. Majority (61.7%) of the food-handlers do not use aprons or wear mask when necessary. Additionally, they eat and drink during working hours. On sanitizer use, 61.7% reported that they do not use sanitizer in washing utensils such as plates, mugs and spoons. All respondents reported that they do not use sanitizer when washing fruits or vegetables. About 83.8% of the institutional food-handlers do prepare meals in advance. Only 17% of respondents reported that they look out for the shelf-life of foods when taking delivery of them.

**Table 5:** Food safety practices among institutional food-handlers

Question	Response % (n)	
	Yes	No
Do you use gloves during the distribution of unpackaged foods? If no, go to question 3.	11.9 (28)	88.1 (207)
Do you wash your hands properly before or after using gloves?	64.2 (18)	35.7 (10)
Do you wear an apron while working?	38.3 (90)	61.7 (145)
Do you wear a mask when you distribute unwrapped foods?	38.3 (90)	61.7 (145)
Do you eat or drink at your work place?	93.6 (220)	6.4 (15)
Do you wear nail polish when handling food?	16.2 (38)	83.8 (197)
Do you prepare a meal in advance (i.e., from one shift to another)?	83.8 (197)	16.2 (38)
Do you properly clean the food storage area before storing new products?	87.2 (207)	12.8 (30)
Do you use the sanitizer when washing service utensils (plates, mugs and spoons)?	61.7 (145)	38.3 (90)
Do you use the sanitizer when washing fruits?	0.0 (0)	100 (235)
Do you check the shelf life of foods at the time of delivery?	17 (40)	83 (195)

Some previous studies suggest that the lack of knowledge in food safety can lead to poor hygienic practices by food-handlers [27, 40]. However, Clayton et al. [41] reported that about 63% of food-handlers demonstrating knowledge in food safety did not demonstrate a corresponding positive behavior towards food safety/hygienic practices. This shows that food-handlers might not necessarily be practicing strict food safety procedures during food handling, even when they provide answers to show that they are knowledgeable in a survey. Therefore, other factors such as employee motivation and continuous education and training on the job should be provided to inspire food-handlers, which will affect attitudes and subsequently food-safety practices [42].

### Logistic regression analyses

Logistic regression analysis testing four models to identify the variables that impacted on the observed results are shown in Table 6. All models tested except model 2 showed statistically significant differences (*p* < 0.05). The significant differences were only observed for models in which the explanatory variable was the level of education. Thus with the exception of model 2, respondents having advanced specific knowledge demonstrated significantly better understanding of food safety issues. For example, in model 1, the results indicate that food-handlers that demonstrate good knowledge of Hepatitis A virus as a food-borne pathogen had an adjusted OR of 14.3 (CI_95_, *P* = 0.002). Thus, these food-handlers have 14.3 more times possibilities of having good level of food safety knowledge when compared with food-handlers lacking sufficient level of knowledge for the same variable.

**Table 6:** Logistic regression analyses

Variables	OR adjusted	*P*-value
Model 1		
Knowledge about risk group for food-borne disease	14.2	0.001
Knowledge about HIV as a food-borne disease	8.4	0.004
Knowledge about Hepatitis virus as a food-borne pathogen	14.3	0.002
Knowledge about Staphylococcus as a food-borne pathogen	3.9	0.017
Knowledge about Clostridium as a food-borne pathogen	7.6	0.002
Knowledge about microorganisms versus health of food handlers	13.2	0.003
Level of education	5.1	0.000
Model 2		
Knowledge of gloves use	0.8	0.104
Knowledge of masks use	1.3	0.052
Knowledge about eating or drinking during working hours	1.4	0.080
Practice about eating or drinking during working hours	2.5	0.063
Level of education	4.2	0.030
Model 3		
Knowledge about freezing versus microorganisms	3.8	0.003
Knowledge about refrigeration temperature	3.5	0.001
Level of education	3.9	0.004
Model 4		
Knowledge about food contamination versus attributes of food that may indicate contamination	6.4	0.010
Knowledge of shelf-life	3.5	0.003
Level of education	4.5	0.001

## Conclusions

In general, institutional food-handlers in Ghana had satisfactory knowledge in the areas of food safety, general and personal hygiene, cleaning and sanitation procedures. However, this did not translate into strict food hygiene practices. Therefore continuous food safety education and motivation for food-handlers of various demographic backgrounds with special attention paid to those with lower levels of education would complement other interventions that pursue the enhancement of food safety systems in Ghana.

## Abbreviations

AIDS: Acquired immune deficiency syndrome
HACCP: Hazard analysis critical control point
JHS/JSS: Junior high school/junior secondary school
KAP: Knowledge attitude and practice
SHS/SSS: Senior high school/senior secondary school
WHO: World health organization
